# Effects of low-dose ionizing radiation on genomic instability in interventional radiology workers

**DOI:** 10.1038/s41598-023-42139-5

**Published:** 2023-09-19

**Authors:** Dominika Kochanova, Sachin Gulati, Matus Durdik, Lukas Jakl, Pavol Kosik, Milan Skorvaga, Katarina Vrobelova, Katarina Vigasova, Eva Markova, Dusan Salat, Andrej Klepanec, Igor Belyaev

**Affiliations:** 1 Department of Radiobiology, Cancer Research Institute, Biomedical Research Center, Slovak Academy of Sciences, Dubravska cesta 9, 845 05 Bratislava, Slovakia; 2 Faculty of Health Sciences, University of Ss. Cyril and Methodius in Trnava, Namestie J. Herdu 577/2, 917 01 Trnava, Slovakia; 3 Institute of Radiation Protection, Ltd., Stanicna 1062/24, 911 05 Trencin, Slovakia; 4 Faculty of Medicine, Comenius University, Spitalska 24 , 813 72 Bratislava, Slovakia

**Keywords:** Chromosomes, DNA damage and repair, Cancer prevention, Chromosomes, Genetics, Health occupations, Risk factors

## Abstract

Interventional radiologists are chronically exposed to low-dose ionizing radiation (IR), which may represent a health risk. The aim of the present study was to evaluate genomic instability by analyzing chromosomal aberrations, micronuclei, and 53BP1 DNA repair foci in peripheral blood lymphocytes of radiologists. Based on the IAEA guidelines on biodosimetry using dicentrics, the average protracted whole-body dose in radiologists were estimated. Since preleukemic fusion genes (PFG) are the primary events leading to leukemia, we also studied their presence by RT-qPCR and FISH. No significant difference in 53BP1 foci and incidence of PFG (MLL-AF4, MLL-AF9, AML1-ETO, BCR-ABL p190) was found in cells of interventional radiologists in comparison to controls. However, our results showed an increased frequency of micronuclei and various types of chromosomal aberrations including dicentrics in interventional radiologists. The average protracted whole body estimated dose was defined at 452.63 mGy. We also found a significantly higher amplification of the MLL gene segment and increased RNA expression in cells of interventional radiologists in comparison to controls. In conclusion, our results showed that long-term low-dose IR induces genomic instability in interventional radiologists.

## Introduction

Radiography, computed tomography, and other diagnostic techniques, which are based on utilizing ionizing radiation (IR), are essential basic tools in a wide range of medical diagnostic procedures and treatments. Despite applied precautions, radiologists are chronically exposed to low-dose IR, which may represent a health risk^1^. The most common health problems associated with long-term exposure of radiologists to low-dose IR are the occurrence of cataracts, dysfunction of the central nervous system, circulatory disease, cardiovascular disease, and, last but not least, cancer^2^. Epidemiological studies have consistently shown that prolonged exposure to low-dose IR not only increased the risk of solid cancers but also elevated risks to develop leukemias^3^.

The origin of leukemia is often conditioned by the creation of preleukemic fusion genes (PFG) as the first hit in leukemogenesis^4^. PFG are formed from chromosomal aberrations (CA), which arise through misrepaired DNA double-strand breaks (DSB)^5^.

The effect of low-dose IR on DNA damage and genomic instability in radiology workers has previously been analyzed by several scientific groups. These studies evaluated: (i) DNA damage using either the comet assay^6^ or the γH2AX/53BP1 DNA repair foci assay^7^, (ii) micronuclei (MN) using the cytokinesis-block micronucleus (CBMN) assay^8–14^, (iii) CA using the dicentric assay^12, 15–17^. Of note, these studies either used only one biomarker^6, 7, 10, 11, 14^ or analyzed CA in a maximum of 200 metaphases^12, 15–17^, which is not sufficient for detecting the effect of the low-dose IR^18^. While several studies reported induction of RNA expression by high doses and low-doses of IR in vitro^19, 20^, only one study identified induction of mRNA expression in medical workers exposed to low-dose X-rays^15^. Considering the reported effects of long-term low-dose IR exposure in radiologists, we decided to perform a complex analysis of DNA damage and genomic instability using a combination of relevant techniques (53BP1 foci assay, CA assay, CBMN, FISH, and RT-qPCR) in cells of interventional radiologists in comparison to unirradiated medical workers.

## Results

### 53BP1 foci

**Figure 1:**
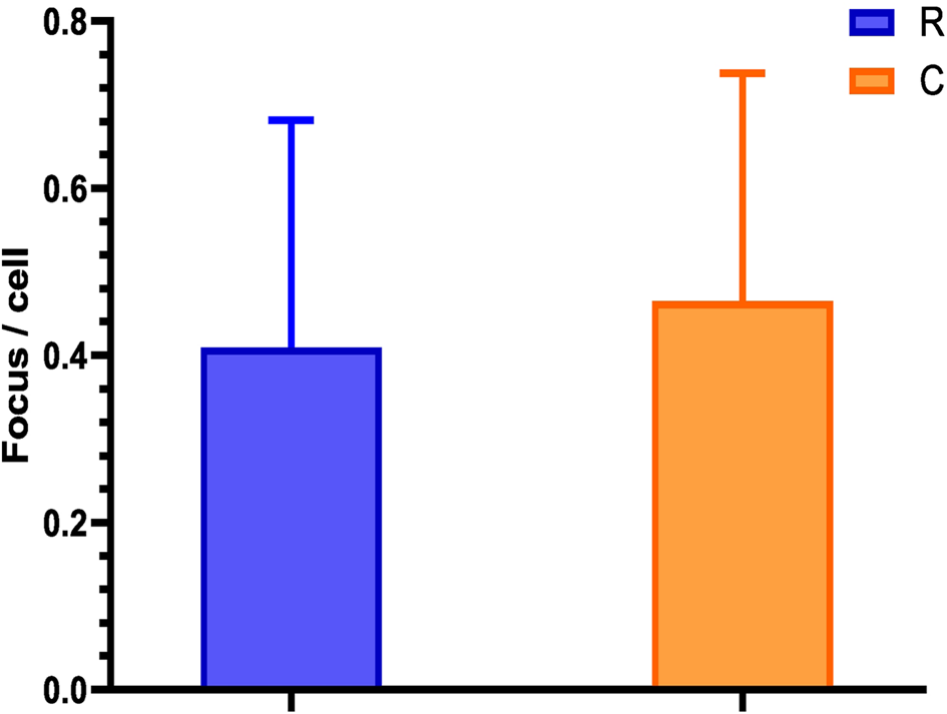
53BP1 DNA repair foci in radiology workers (R) and negative controls (C). Data are presented as mean ± SD.

We examined DSB in peripheral blood lymphocytes (PBL) by quantification of fluorescently-labeled 53BP1 foci using imaging flow cytometry (IFC). The mean number of 53BP1 foci in the cells of radiologists was 0.41 foci per cell, while the mean number in the cells of controls was 0.47 foci per cell. Statistical analysis showed no difference in the 53BP1 DNA repair foci production between interventional radiology workers and controls (univariate ANOVA; p = 0.507728) (Fig. 1).

### Micronuclei

**Figure 2:**
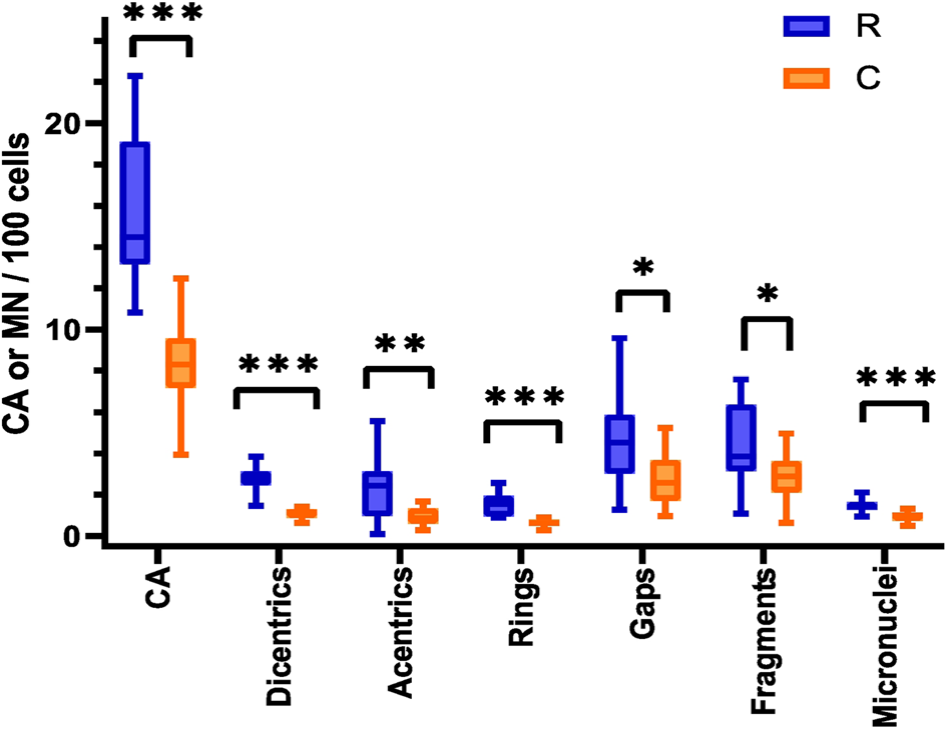
Chromosomal aberrations (CA) and micronuclei (MN) in PBL of radiologists (R) in comparison to controls (C). The frequency of total aberrations (CA), dicentrics, acentrics, ring chromosomes, chromatid gaps and chromatid fragments was analyzed. Statistically significant differences are shown as revealed by the ANOVA with the Fisher LSD at p < 0.05 (*), 0.01 (**) and 0.001 (***). All data are presented as mean ± SD.

The frequency of micronuclei per binucleated cells was significantly higher in the cells of the radiology workers than in the control group (univariate ANOVA; p = 0.000040) (Fig. 2). Based on the obtained results, we conclude that long-term exposure to low-doses of IR can induce formation of micronuclei in the PBL of interventional radiologists.

### Chromosomal aberrations

By using univariate ANOVA we found a significantly higher frequency of rings (p < 0.00001), dicentrics (p < 0.00001), chromatid gaps (p = 0.012850), chromatid fragments (p = 0.020689), acentric chromosomes (p = 0.003081), and total aberrations (p < 0.00001) in the samples of the radiological workers (Fig. 2). The CA results confirmed our findings obtained using the CBMN assay and suggested a higher accumulation of chromosomal damage in the radiological workers in comparison to the control group.

### MLL rearrangements

**Figure 3:**
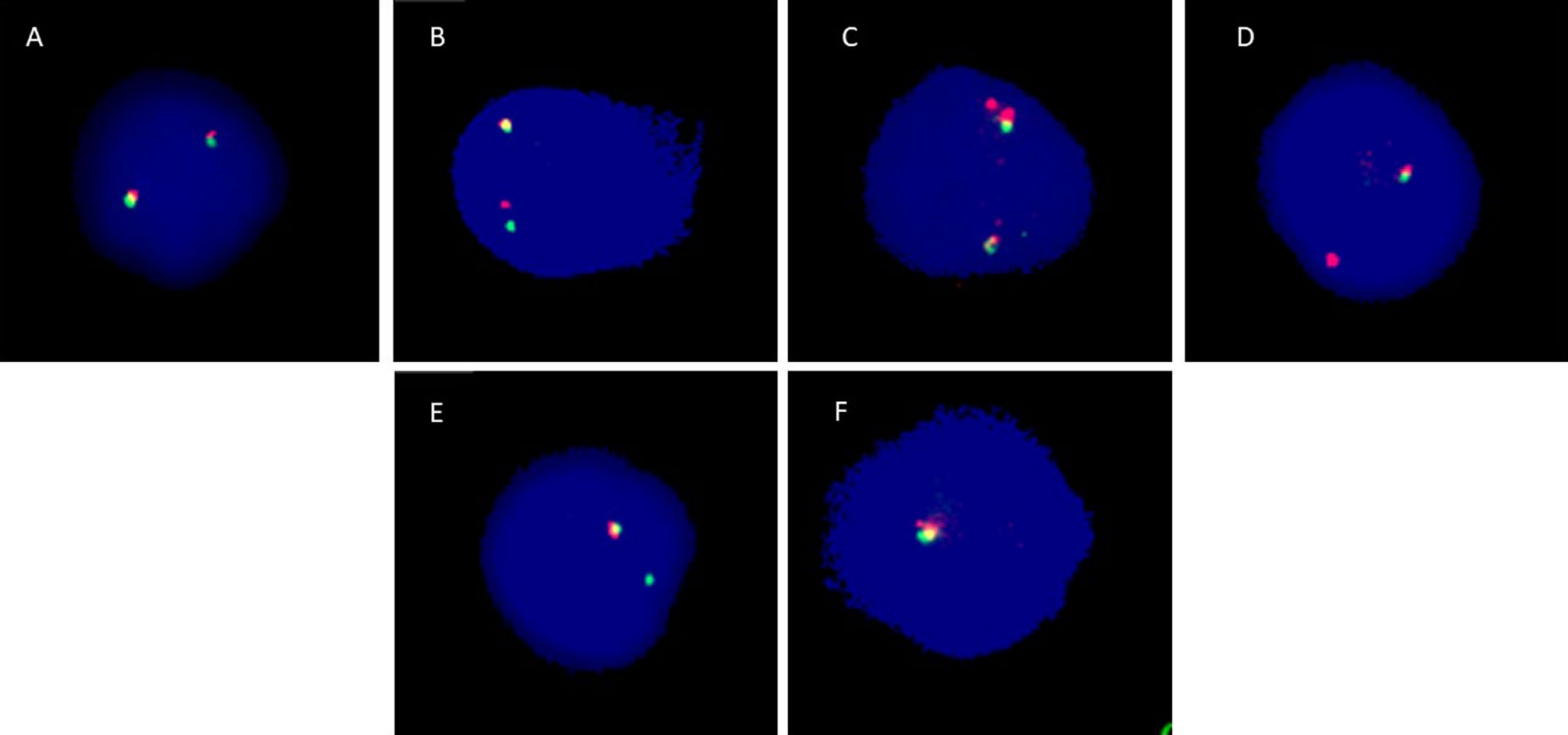
Representative images of the cell nuclei, which were obtained by the FISH analysis using the MLL breakapart probe. (**A**) Cell contains two intact MLL genes, each visualized with co-localization of green and red signals, which mark two parts of the MLL gene. While the upstream (red) signal represents the MLL segment between the breaking point and gene PHLDB1 (Pleckstrin homology like domain family B member 1) closer to the telomere, the MLL segment between UBE4A gene (Ubiquitination factor E4A) and breaking point closer to the centromere is shown by green staining (downstream signal). (**B**) Cell with translocation of the MLL gene, which is visualized as one red (upstream) and one green (downstream) signal. (**C**) Cell with duplication of the red (upstream) signal, which is manifested by the presence of one redundant red signal. The MLL deletion is visualized by loss of the green (downstream) signal (**D**), red (upstream) signal (**E**) or whole MLL gene (**F**).

**Figure 4:**
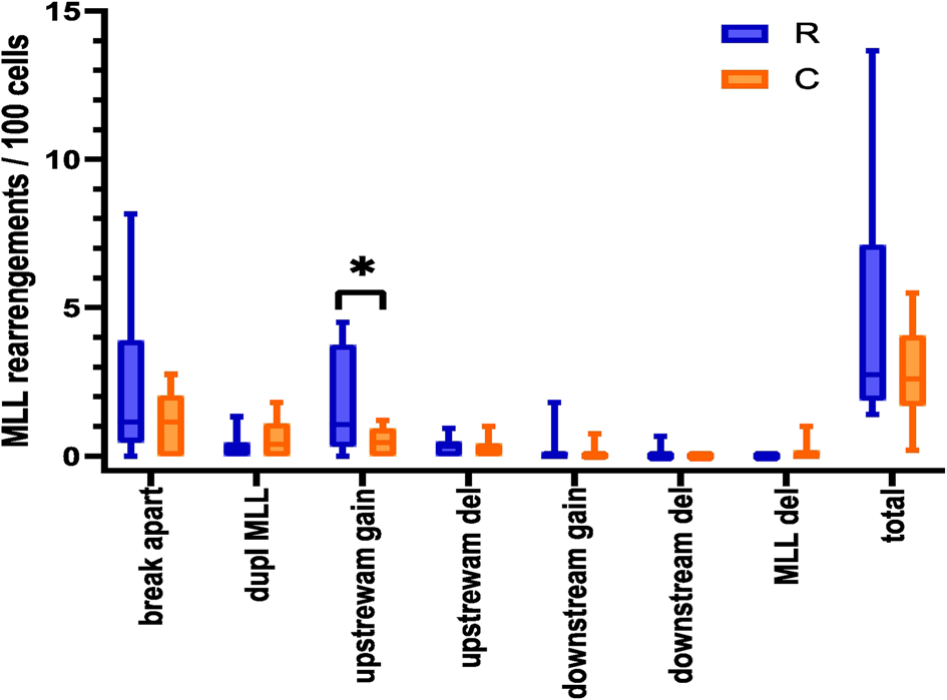
MLL rearrangements in PBL of radiologists (R) and controls (C). The graph represents MLL translocations (Trans MLL), duplications of the MLL gene (Dup MLL), gains of the upstream/downstream MLL segment (upstream/downstream gain), deletions of the upstream part/downstream part of the MLL gene (upstream/downstream del), deletion of whole MLL gene (Del MLL) and total rearrangements of the MLL gene (Total) in the radiologists examined groups. Statistically significant differences are shown as revealed by Mann–Whitney *U* test at p < 0.05 (*).

The MLL (KTM2A, histone-lysine N-methyltransferase 2A) gene is a key regulator of transcription in hematopoiesis, and an alteration of its function could be a risk factor in the development of leukemia. The MLL gene rearrangements were analyzed using a breakapart FISH probe (Fig. 3), which can detect translocations, deletions, and duplications of the MLL gene. No statistically significant differences in the frequency of MLL translocations, duplications, gains of the downstream segment of the MLL gene, deletions of the upstream part of the MLL gene, deletions of the downstream part of the MLL gene, deletions of whole MLL gene, and total rearrangements of the MLL gene were detected (p > 0.05). However, we observed a significantly increased incidence of gains of upstream segment of the MLL gene in interventional radiology workers (p = 0.047) (Fig. 4). In conclusion, long-term low-dose exposure to IR may lead to the amplification of the upstream segment of MLL gene and potentially contributing to malignant transformation^21^.

### Preleukemic fusion genes

Using the RT-qPCR method, we quantified RNA of the blood cells for the occurrence of PFG in the radiologists and the control group. RT-qPCR revealed the presence of MLL2-AF4 in 8/26 (30.8%), MLL-AF9 1/26 (3.8%), AML1-ETO 11/26 (42.3%), BCR-ABL 3/26 (11.5%). Sequencing of the qPCR products (the validation coefficient being ~ 87%) revealed a false positivity only in three MLL fusion genes, namely two for MLL-AF4 and one for MLL-AF9. In summary, we got 6/26 positive samples for MLL2-AF4 (23%), 0/26 for MLL-AF9 (0%), 11/26 for AML1-ETO (42.3%) and 3/26 for BCR-ABL (11.5%). While we observed three positive samples for MLL2-AF4, six for AML1-ETO, and two for BCR-ABL in radiologists, we also found three positive samples for MLL2-AF4, five for AML1-ETO and one for BCR-ABL in controls. We did not detect increased abundance of PFG in the cells of radiologists. Interestingly, statistical analysis revealed a significantly higher amount of total RNA per cell among radiologists as compared with the control group (univariate ANOVA, p = 0.000057) (Table S1). The quality of isolated RNA was validated by the c-Abl control gene copy number per 10^5^ cells and did not differ between the groups (p > 0.05) (Table S1). In conclusion, long-term exposure to low-dose IR does not affect the occurrence of tested PFG but can induce RNA expression.

### Association between years of practice and 53BP1/CA/MN/MLL rearrangements

We performed a linear regression analysis to determine the possible effect of duration of radiological practice on DNA damage and genomic instability. We did not obtain any significant association between years of practice and any of the studied biomarkers—53BP1 foci, CA, MN and MLL rearrangements (p > 0.05) (Table S2). The lack of correlation between the years of practice and the measured biomarkers is likely related to the limited lifespan of lymphocytes (530–1600 days) and their clearance from the blood system^22^.

### Association between age of participants and 53BP1/CA/MN/MLL rearrangements

Linear regression was also used to examine of possible effect of age. The age of participants did not affect the frequency of 53BP1 foci, MN, CA, and MLL rearrangements (p > 0.05) (Table S2). This result can be accounted for the limited age range of the participants enrolled in the study.

### Protracted whole body dose estimation

**Figure 5:**
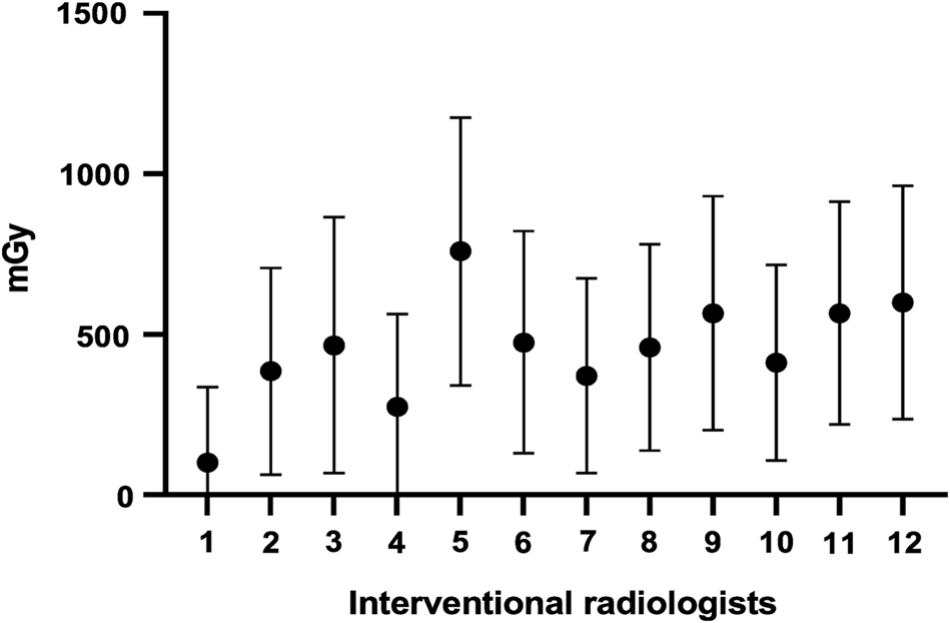
Protracted whole body estimated dose in interventional radiologists. Data were obtained using equation (Y = C + αD) recommended by the IAEA for biodosimetry based on dicentrics^18^ with linear coefficient (α) from Llyod et al.^23^. Data are presented as mean ± CI.

Only fragmentary data on physical individual dosimetry were available for the interventional radiologists enrolled in this study, which covered a limited duration of their practice (not shown). As far as dicentrics are considered to be a gold standard for biodosimetry, we assessed the absorbed doses for the interventional radiologists using the obtained data on dicentrics according to the equation recommended by the IAEA^18^ and linear coefficient for X-rays exposure from Llyod et al.^23^. While the individual protracted whole-body doses varied from 100.15 to 752.63 mGy, the mean dose was found to be 452.63 mGy (Fig. 5).

## Discussion

The aim of this study was to investigate DNA damage and genomic instability in the PBL of interventional radiologists by using a combination of relevant techniques. DSB was analyzed by quantifying fluorescently labeled 53BP1 DNA repair foci, which mark unrepaired or incorrectly repaired DSB and may persist for some time after irradiation. Our results showed no increase in the amount of the DNA repair foci in lymphocytes of the interventional radiology workers as compared to the controls. This is in line with the results by Barsi et al. who did not observe any significant increase of 53BP1 foci in the PBL of the radiology workers^7^. While the previous study used fluorescence microscopy^7^, we analyzed the 53BP1 foci by the IFC in our experiments. On the other hand, low-dose IR exposure of the radiologists was chronic and the accumulated dose was estimated to be higher than 50 mGy for all the interventional radiologists (Fig. 5). The obtained negative results may be caused by the relatively short lifespan of the DNA repair foci, which do not accumulate in vivo following repeated irradiations^24^.

We enriched the dicentric assay with analysis of other chromosomal aberrations, such as ring chromosomes, chromatid gaps, chromatid fragments, and acentric chromosomes. In contrast to some previous reports^12, 15–17^, which analyzed maximum of 200 metaphases, and according to the IAEA guidelines for biodosimetry of the low-dose IR^18^, we evaluated 1000 metaphases per sample. To complement the CA assay, we also examined MN using the CBMN assay. In our control samples, the frequency of MN did not differ from the data of healthy population as previously reported^25^. Frequency of dicentrics + rings in our control sample is in line with results from Carbonell et al.^26^, but slightly higher in comparison to majority of publications^27, 28^. The main reason for this difference could be that our control samples came from various hospital departments where medical staff might be exposed to different carcinogenic and/or genotoxic agents such as formaldehyde, organic solvents, anesthetic gases, and anticancer drugs. Chronic exposure to anesthetic gases or formaldehyde could significantly increase the level of chromosomal aberrations, including dicentric chromosomes^29, 30^. Musak et al. also found an increased frequency of chromatid-type, chromosome-type (including dicentrics), and total chromosomal aberrations in medical workers from Slovakia after chronic exposure to volatile anesthetics, antineoplastic agents, and formaldehyde^31^.

However, we found a significantly increased number of dicentrics, ring chromosomes, acentrics, chromatid gaps, and chromatid fragments in the lymphocytes of radiological workers. In line with this data, our results also showed that the MN frequency was significantly higher in radiologists as compared to controls. These observations are also in line with several other studies, which demonstrated a significantly higher level of CA and MN in radiology personnel^8, 11, 15, 16^. Of note, these previous studies are heterogeneous in several aspects. In particular, the types of analyzed CA are not always clearly defined and their frequencies vary between studies. In addition, the incidence of MN and CA can be affected by various confounders such as age of participants^11, 12, 16, 32^. To determine possible effect of age, we performed a regression analysis, which did not show an association between age and any of the analyzed biomarkers (53BP1 foci, CA, MN, and PFG) in our study groups. This result can be accounted for the age range of our participants (from 31 to 68 years – interventional radiologists; from 30 to 64 years – controls), which was not similar to that in those previous studies^11, 12, 16, 32^, where the age dependence of MN and CA was shown. Smoking or alcohol consumption has also been reported as a confounding factor for genomic instability^13, 33^. In our study, we eliminated the effect of smoking and alcohol consumption by selecting appropriate participants, which were all occasional alcohol users and non-smokers (except for one participant). Available studies indicate that the sex of participants can be another common confounder^12, 34, 35^. In particular, a higher frequency of MN was found in women as compared with men using the MN assay^35^. We did not observe any sex related differences when comparing radiology and control groups. The number of females in the studied groups was too low in order to perform a proper statistical analysis, which would associate the impact of low-dose IR on DNA damage and sex-related differences.

In leukemogenesis, chromosome rearrangements are often formed due to PFG, which can cause the formation of preleukemic clones and eventually overt leukemia. We investigated four PFG, namely: MLL-AF4, BCR-ABL p190, and AML1-ETO, MLL-AF9, which are characteristic for both acute lymphoblastic leukemia (ALL) and acute myeloid leukemia (AML), respectively. In our study, PFG were identified by RT-qPCR and results were validated by sequencing. Overall, 33.3% of radiologists and 57.1% of control subjects did not carry any of monitored PFG associated with leukemia. However, the incidence of studied PFG did not differ between radiologists and controls. The incidence of BCR-ABL p190 and AML1-ETO in our control participants did not differ from incidence of these two PFG (0–80% for BCR-ABL p190 and 19.5% to 22% for AML1-ETO) in healthy population^36–38^. On the other hand, our control participants had a decreased number of MLL-AF4 (11.5%) in comparison to healthy population (52%) as previously reported^37^. This difference can be accounted for lower statistical power and possible cross contamination in the previous study. To our knowledge, no study has analyzed the incidence of MLL-AF9 in adults.

We have previously analyzed PFG after acute in vitro low-dose irradiation (≤ 500 mGy) of lymphocytes, human hematopoietic stem, and progenitor cells from umbilical cord blood^39, 40^. A significant increase of BCR-ABL was observed in irradiated cells. However, other PFG (TEL-AML1, MLL-AF4, AML1-ETO, MLL-AF9, or PML-RARA) were not induced by irradiation^39, 40^. Possible reason for data inconsistencies between the in vitro and in vivo studies can be either the source of cells or the irradiation method. While in our present study we isolated cells from fresh blood of persons who are chronically exposed to radiation, the aforementioned in vitro studies used frozen-thawed umbilical cord blood cells and acute in vitro irradiation.

In the MLL gene, more than 135 different rearrangements have been identified, of which more than 94 translocation partner genes were characterized at the molecular level^41^. This gene is also known to generate duplications, deletions, or amplifications resulting in leukemia^42^. To track the entire MLL locus, we used a breakapart FISH probe covering an 87 kb region telomeric to the MLL gene and a 170 kb region centromeric to the MLL gene^43^. This MLL probe offers high specificity and the advantage of detecting not only PFG but also other chromosomal rearrangements such as amplification (so called gains of the signal), duplications of the whole gene, and deletions. We report here for the first time on statistically significantly increased amplification of upstream signals in cells of interventional radiologists. By the MLL breakapart probe constitution, the gains of upstream signal express the amplification of the telomeric region of the MLL gene and noncoding sequence including SHGC-111513 marker located telomeric to the MLL gene. The length of the amplified sequences is not defined, because it can also include a DNA sequence containing PHLDB1 gene^43^. The mutations of PHLDB1 gene have been observed in different types of cancer, especially several gliomas^21^.

We also report here a higher amount of RNA per cell in interventional radiologists in comparison to controls. One possible explanation could be that ionizing radiation affects the expression of non-coding RNA (ncRNA), which represents 98% of the human genome^44^. Some authors detected upregulation or downregulation of ncRNA transcription as mRNA, circular RNA or long ncRNA (lncRNA) after exposure to low-doses of IR in various cells^19, 20^. For example, Mikhailov et al. demonstrated downregulation of mature miRNA expression, specifically miR-27a and miR-181, as well as upregulation of expression of some lncRNAs—MALAT1 and GAS5^20^. These ncRNA are involved in the regulation of p53 transcription factor pathway^20^. Our data on increased RNA expression in cells of interventional radiologists warrants further studies to validate the nature of the affected RNA fraction.

While some studies reported on a correlation between the levels of DNA damage, MN, CA and years of practice^6, 12^, our analysis did not reveal such a correlation between years of practice and any of the analyzed biomarkers. Such data inconsistency between our and other studies^6, 12^ can be accounted for different mean years of practice. The extinction of lymphocytes due to their limited lifetime, which is estimated in the range from 530 to 1600 days^45^, can have an influence on the correlation between the analyzed biomarkers and the duration of work^22^. However, the lifetime of lymphocyte only concerns DNA damage and genomic instability induced by radiation occurring in the exposed lymphocyte itself. This lymphocyte is eliminated whereupon does not in itself pose a leukemic risk. Considering that, our measured biomarkers (53BP1 foci, CA, MN and PFG) in lymphocytes represent only surrogate marker, leukemia risk should be associated with DNA damage and genomic instability in the hematopoietic stem cell. Persistently increased leukemia risk from genomic instability in hematopoietic stem cells should translate to peripheral lymphocytes and remain elevated regardless of lymphocyte lifespan. Other reasons why we did not find a relationship between years of practice and biomarkers could be the lack of information on cumulative dose in studied interventional radiologists, their working area or job rotation during their practice. Of note, Vral et al. have previously reported that radiologists do not always wear their dosimeters correctly^14^. In addition, the correct position of the individual dosimeter under the apron is important for measuring the personal dose.

While only fragmentary data on individual physical dosimetry covering relatively short periods of practice were available, we performed biodosimetric assessment of the IR absorbed dose in occupationally exposed radiologists. For this purpose, the data on dicentrics were used according to the IAEA recommendations^18^. While the general population is exposed to a mean dose of 3 mSv/year from natural sources^46^ and the safety limit for whole body irradiation of professional workers is 50 mSv/year^47^, we found that interventional radiologists, who were occupationally exposed to IR for an average of 18 years, received an average protracted whole body dose of 452.63 mGy.

The majority of publications, that studied medical personnel determined the received annual dose in the range of 0–48 mSv^8, 11, 48^. Our results are closest to the results from Fang et al. who detected that the cumulative effective dose of these workers with the duration of practice 1–31 years ranged from 2.81 to 416.43 mSv^15^. However, Fang et al. measured cumulative effective dose in hospital workers exposed to low-dose IR by personal thermoluminescence dosimeters every 3 months and our publication focused on protracted whole-body dose.

The current study was set up to provide a complex assessment of DNA damage and genomic instability assessment in the peripheral blood lymphocytes of in interventional radiologists who are chronically exposed to low-dose IR. The increased amount of CA, namely: dicentric chromosomes, ring chromosomes, chromatid gaps, chromatid fragments, acentric chromosomes, and micronuclei were found in occupationally exposed interventional radiology workers. To the best of our knowledge, this is the first study, which investigated the incidence of PFG, MLL rearrangements and total RNA expression in radiological workers. While our results showed no induction of the tested preleukemic fusion genes (MLL-AF4, MLL-AF9, AML1-ETO, and BCR-ABL p190) we could demonstrate a significant increased RNA expression segment in cells of interventional radiologists. Thus, harmful effects of long-term low-dose IR exposure were demonstrated in terms of genomic instability, which may be associated with induction of malignancies. On the other hand, the incidence of 53BP1 DNA repair foci was the same in cells of interventional radiologists and control participants. These findings are most probably due to the relatively short expression of DNA repair foci, which do not persist for longer post-irradiation time in contrast to CA and MN. Likewise, we did not find a significant correlation between years of practice and any of the tested biomarkers, which can be explained by the time dependent elimination of lymphocytes. In the future, the hospitals should continue to raise awareness of radiation protection principles, introduction of more frequent preventive examinations or stricter exposure limits among medicine workers exposed to radiation.

## Material and methods

### Participants

This study was approved by the Ethics Committee of the University of Ss. Cyril and Methodius in Trnava and all experiments and methods were performed in line with relevant guidelines. The informed consent was acquired from each participant before the collection of a blood sample. Blood samples were taken from 12 interventional radiologists working in six various hospitals in Slovakia and 14 controls. The radiological group consisted of participants whose profession is associated with chronic exposure to low-dose IR. On the other hand, participants in control group have worked in other hospital departments without IR exposure. There was no difference in sex and age between interventional radiologists (R) and control (C) participants (Table 1). Smoking and alcohol status, infectious diseases, medications, hormonal contraception, recent X-ray examinations were determined by a questionnaire and their distribution were similar between radiology and control groups (Table 1). Individuals with chemotherapy, radiotherapy, or cancer history were excluded from the study.

**Table 1:** Characteristics of the participants according to the questionnaires filled in by the interventional radiologists (R) and control medical workers (C).

Participants	*R*	*C*
Number	12	14
Age		
Mean	45	44
Minimum	31	30
Maximum	68	64
Years of practice with IR		
Mean	18	0
Minimum	5	0
Maximum	42	0
Sex		
Female	1	2
Male	11	12
Smoking		
Yes	1	0
No	11	14
Alcohol		
Yes	0	0
No	0	0
Occasionally	12	14
Medication		
Yes	2	1
No	10	13
Illness		
Yes	2	0
No	10	14
X-rays examination		
Yes	2	0
No	10	14
Hormonal anticonception		
Yes	0	1
No	12	13

### Isolation of blood cells 

Mononuclear cells were isolated from the blood samples of participants by density gradient centrifugation using Lymphocyte Separation Medium (MP Biomedicals, Illkirch-Graffenstaden, France) as described previously^49^. Adherent monocytes were removed by 2 h incubation (5% CO_2_ and 37 °C). The remaining cells, predominantly lymphocytes, were counted in the Burker chamber using 0.4% Trypan blue (Gibco/Thermo Fischer Scientific, Waltham, Massachusetts, United States). Viability of the cells was more than 95%.

### Imaging flow cytometry

**Figure 6:**
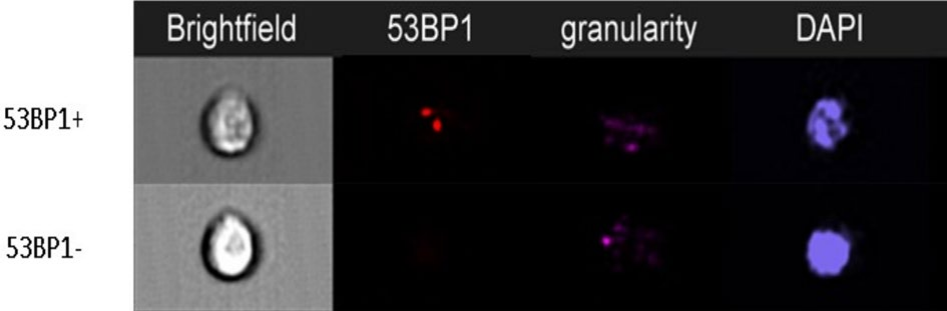
Representative images of PBL obtained by the IFC. Brightfield is visualized by forward scatter, granularity by side scatter, 53BP1 by fluorescence of specific antibody, and DNA.

DNA repair foci in lymphocytes were analyzed by imaging flow cytometry (IFC) using the ImageStreamX-100 (Amnis, Seattle, Washington, United States) as described previously^50^. After isolation, the cells were fixed by cold 3% paraformaldehyde, washed with PBS, permeabilized with cold 70% ethanol and stored in the fridge. The next day, cells were stained using a primary anti 53BP1 monoclonal/mouse antibody (a gift from Prof. Halazonetis (University of Geneva, Switzerland)) in concentration 1:20 and incubated for 2 h at room temperature (RT). Afterward, the secondary Alexa-Fluor 488 goat anti-mouse antibody (Sigma Aldrich, St. Louis, Missouri, United States) in concentration 1:250 was added and incubated for 1 h at RT. Samples were washed in PBS and DNA was stained with 3 µM DAPI (Sigma Aldrich). We analyzed a minimum of 10,000 cells per sample using a 63 × magnification objective along with an extended depth of field for the maximal possible resolution. Three lasers (405, 488, 785 nm) were used to visualize DNA, 53BP1, and granularity, respectively (Fig. 6). 53BP1 foci were quantified by the IDEAS software as previously described^50^.

### Chromosomal aberrations and cytokinesis-block micronucleus assay

**Figure 7:**
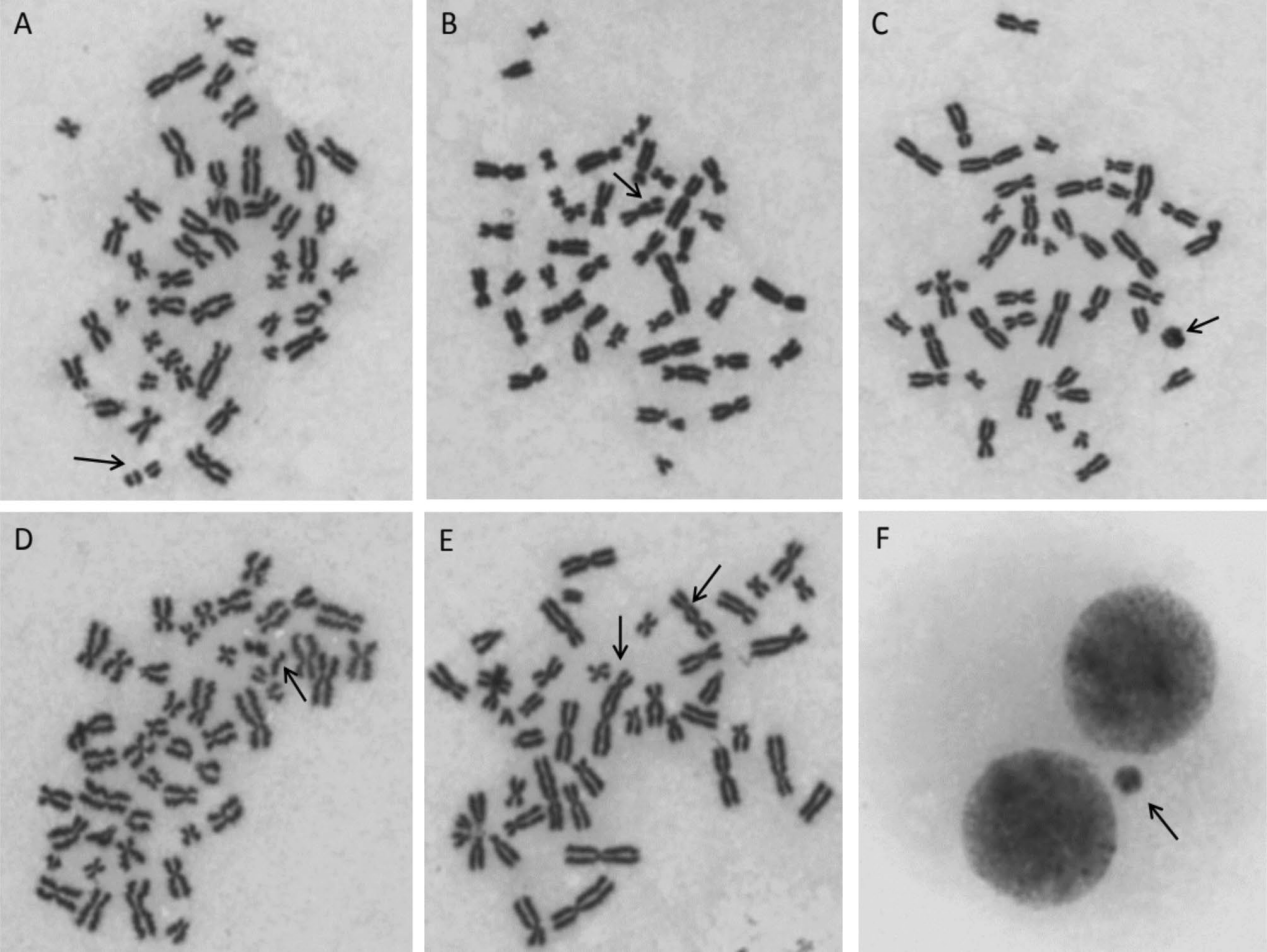
Representative images of metaphases with: acentric chromosome (**A**), chromatid gap (**B**), ring chromosome (**C**), chromatid fragment (**D**), dicentric chromosomes (**E**), and micronucleus (**F**).

Micronuclei and chromosomal aberrations were analyzed according to Fenech et al. and Moorhead et al.^51, 52^ as previously described^53^.One thousand well-spread metaphases were automatically detected and further analyzed for CA in a double-blinded manner using the Zeiss Axio Imager Z2 fluorescence microscope (Carl Zeiss, Oberkochen, Germany) and the Metafer 3.6 software (MetaSystems, Altlusheim, Germany). The selection of metaphase and criteria for cytogenetic abnormalities complied to the generally accepted recommendations^54^. Dicentrics, ring chromosomes, chromatid gaps, chromatid fragments and chromosome fragments (acentric chromosomes) were manually analyzed (Fig. 7). To determine the frequency of binucleated cells with MN, a total of 1000 binucleated cells were scored for each sample.

### RNA isolation, cDNA synthesis and RT-qPCR

Total RNA was isolated with innuPREP DNA/RNA Mini Kit (Analytik Jena AG, Jena, Germany). The concentration of RNA was measured by Nanodrop (Thermo Scientific, St. Leon-Rot, Germany). The 1 µg of total RNA was reversely transcribed into cDNA using standard protocol with random hexamers and oligo(dT)18 as recommended by the manufacturer (ThermoFisher Scientific, St. Leon-Rot, Germany). RT-qPCR was performed according to Gabert et al.^55^ with the following modifications: (i) final volume: 20 µl, (ii) template: 2 µl cDNA, (iii) master mix: 4 µl 5 × HOT FIREPol Probe qPCR Mix Plus (ROX), (iv) PCR cycling conditions: 1 cycle: 95 °C 12 min, 45 cycles: 95 °C 15 s, 60 °C 1 min by AriaMX real-time PCR system (Agilent Technologies, Santa Clara, California, United States).

The primers and probes were designed according to Gabert et al., Jansen et al., Beillard et al.^55–57^ and were synthesized by VBC-Biotech (Vienna, Austria) (Table S1). Individual fusion genes subcloned into PCR II TOPO vectors were used as plasmid standards (Qiagen, Marseille, France). Frequently occurring PFG associated with ALL and AML, including MLL-AF4, MLL-AF9, AML1-ETO and BCR-ABL p190 were tested. The samples have been considered as positive when at least one reaction of a triplicate was tested positive.

### Validation of the RT-qPCR data

The positivity of the sample for PFG was confirmed by a two-stage validation, namely by: (i) amplification of qPCR product with PFG specific primers in a standard PCR, (ii) sequencing of amplified PCR products. The PCR product after re-amplification was subcloned into a sequencing vector and recombinant plasmid DNA was employed as a template in the sequencing reaction using sequencing primers of the vector. Sequencing was performed using BigDye® Terminator v3.1 Cycle Sequencing Kit, following manufacturer’s protocol (Applied Biosystems, Austin, TX, United States).

### Fluorescence in situ hybridization (FISH)

Lymphocytes were fixed on a polysine slide and hybridized with a directly labeled MLL breakapart probe (CytoCell Aquarius, Cambridge, United Kingdom) as described previously^58^. At least 100 cells from each sample were manually analyzed using fluorescent microscopy (Olympus BX51, Shinjuku, Japan) at the magnification of 100 × in spectrum red, spectrum green, and spectrum blue (Fig. 4). Representative images acquisition from FISH analysis were made using the METAFER Slide Scanning System based on Zeiss Axioscop 2 epifluorescent microscope, main parameters being: 63x- objective magnification, 10 number of focus planes, and 28/40 m focus plane distance.

### Protracted whole body dose estimation

For evaluating the minimal possible protracted whole-body dose in radiologists the Eq. (1) was applied:

1 $$\mathrm{D}=\frac{\mathrm{Y}-\mathrm{C}}{\mathrm{\alpha }},$$

where Y represents the yield of dicentrics, C is the control yield of dicentrics (0.0109), α is the linear coefficient (0.0364) and D is the dose. The Eq. (1) was set according to the International Atomic Energy Agency (IAEA) recommendations^18^ and linear coefficient for X-rays exposure was derived from Llyod et al.^23^.

### Statistical analysis

The normality of data distribution was tested by the Shapiro–Wilk test. While the data from FISH analysis were not normally distributed and thus evaluated by Mann–Whitney *U* test, the normally distributed data from DSB, CA, MN and PCR assays were analyzed by the factorial analysis of variance (ANOVA) with the Fisher LSD, using Statistica 8.0 software (Dell software, Round Rock, Texas, United States), Prism GraphPad 8.4.3 software and Microsoft Excel. The results were considered significantly different at p < 0.05. If the p-value was lower than 1 × 10^–5^ we displayed the p-value as p < 0.00001. Linear regression analysis Y = α + βD was used for the verification of the possible relationship between each of analyzed biomarkers and years of practice/age. It was validated by the R2–the coefficient of determination. The dose estimation was calculated using Biodose Tools^59^ and confidence intervals evaluated by Delta method.

### Supplementary Information

Supplementary Tables.

## Data Availability

The datasets used and/or analyzed during the current study available from the corresponding author on reasonable request.
