# Supplementary material for: Effects of low-dose ionizing radiation on genomic instability in interventional radiology workers
Source: Sci Rep. 2023 Sep 19;13:15525. doi: 10.1038/s41598-023-42139-5 (PMC10509213; doi:10.1038/s41598-023-42139-5)
Supplement: Supplementary file 1 — Supplementary Tables. [file 41598_2023_42139_MOESM1_ESM.docx]

**Supplementary Table 1.** Results of the RT-qPCR and sequencing analysis of peripheral blood lymphocytes from radiologists (R) and controls (C). The table shows the amount of RNA/cell, number of cells, indicator of quality of isolated RNA, c-Abl control gene copy, number of PFG (MLL-AF4, MLL-AF9, BCR-ABL p190, AML1-ETO) by RT-qPCR and sequencing. * - represents results from RT-qPCR, which have been validated by the sequencing analysis.

|  |  |  |  |  |  |  |  | **Summary**  **translocation No.** | | | |  |  |
| --- | --- | --- | --- | --- | --- | --- | --- | --- | --- | --- | --- | --- | --- |
|  | **RNA yield (pg/cell)** | **Cell No. x 10^6^** | **c-Abl**  **(10^5^ cell eq)** | **MLL-AF4** | **MLL-AF9** | **BCR-**  **ABL p190** | **AML1-ETO** | **by RT-qPCR** |  | **seq**  **validated*** | | |  |
| R | 1.37 | 4.5 | 65.200 | 0/3 | 0/3 | 0/3 | 1/3* | 1/4 |  |  | 1/1 | | |
| R | 0.97 | 5.0 | 50.200 | 0/3 | 0/3 | 0/3 | 0/3 | 0/4 |  |  | - | | |
| R | 1.23 | 3.2 | 32.900 | 0/3 | 0/3 | 0/3 | 0/3 | 0/4 |  |  | - | | |
| R | 0.67 | 4.8 | 25.600 | 0/3 | 0/3 | 0/3 | 1/3* | 1/4 |  |  | 1/1 | | |
| R | 1.01 | 4.0 | 116.000 | 1/3* | 1/3 | 2/3** | 0/3 | 3/4 |  |  | 2/3 | | |
| R | 0.96 | 4.0 | 57.800 | 0/3 | 0/3 | 1/3* | 0/3 | 1/4 |  |  | 1/1 | | |
| R | 1.11 | 4.0 | 96.600 | 0/3 | 0/3 | 0/3 | 0/3 | 0/4 |  |  | - | | |
| R | 1.20 | 4.0 | 60.900 | 0/3 | 0/3 | 1/3* | 0/3 | 1/4 |  |  | 1/1 | | |
| R | 1.04 | 4.5 | 37.500 | 1/3* | 0/3 | 1/3* | 0/3 | 1/4 |  |  | 1/1 | | |
| R | 0.98 | 3.5 | 23.500 | 0/3 | 0/3 | 0/3 | 0/3 | 0/4 |  |  | - | | |
| C | 0.53 | 5.0 | No cq | 0/3 | 0/3 | 0/3 | 0/3 | 0/4 |  |  | - | | |
| C | 0.60 | 5.0 | 14.900 | 1/3* | 0/3 | 1/3* | 0/3 | 2/4 |  |  | 2/2 | | |
| R | 0.55 | 4.0 | 33.900 | 1/3* | 0/3 | 1/3* | 0/3 | 2/4 |  |  | 2/2 | | |
| R | 0.98 | 4.0 | 40.400 | 1/3 | 0/3 | 1/3* | 0/3 | 2/4 |  |  | 1/2 | | |
| C | 0.67 | 1.5 | 16.900 | 0/3 | 0/3 | 0/3 | 0/3 | 0/4 |  |  | - | | |
| C | 0.64 | 4.0 | 31.300 | 0/3 | 0/3 | 1/3* | 0/3 | 1/4 |  |  | 1/1 | | |
| C | 0.57 | 4.0 | 29.800 | 1/3 | 0/3 | 1/3* | 3/3*** | 3/4 |  |  | 2/3 | | |
| C | 0.64 | 4.0 | 44.500 | 0/3 | 0/3 | 1/3* | 0/3 | 1/4 |  |  | 1/1 | | |
| C | 0.85 | 5.0 | 29.600 | 1/3* | 0/3 | 1/3* | 0/3 | 2/4 |  |  | 2/2 | | |
| C | 0.80 | 4.5 | 52.200 | 0/3 | 0/3 | 0/3 | 0/3 | 0/4 |  |  | - | | |
| C | 0.85 | 5.44 | 55.000 | 0/3 | 0/3 | 0/3 | 0/3 | 0/4 |  |  | - | | |
| C | 0.69 | 5.03 | 40.900 | 0/3 | 0/3 | 0/3 | 0/3 | 0/4 |  |  | - | | |
| C | 0.44 | 5.0 | 35.700 | 0/3 | 0/3 | 0/3 | 0/3 | 0/4 |  |  | - | | |
| C | 0.42 | 5.0 | 63.200 | 0/3 | 0/3 | 0/3 | 0/3 | 0/4 |  |  | - | | |
| C | 0.72 | 5.0 | 65.300 | 0/3 | 0/3 | 0/3 | 0/3 | 0/4 |  |  | - | | |
| C | 0.76 | 5.0 | 82.500 | 1/3* | 0/3 | 0/3 | 0/3 | 1/4 |  |  | 1/1 | | |

**Supplementary Table 2.** Linear regression analysis of relationship between years of practice of radiologists/age of all participants form one side and micronuclei/chromosomal aberrations/MLL gene rearrangements from another side. No regression analysis between deletions of the whole MLL gene and years of practice was provided due to the lack of MLL deletions (0%) in the radiology group. Coefficient of determination (R2) along with β variable and p-value is shown.

|  | **Age (all probands)** | **Years of practice (radiologists)** |  |
| --- | --- | --- | --- |
| MN | R2 = 0.015598 | R2 = 0.020331 |  |
|  | β = 0.005206 | β = 0.004697 |  |
|  | p = 0.543255 | p = 0.658442 |  |
| Total CA | R2 = 0.004639 | R2 =0.001130 |  |
|  | β = 0.035686 | β = 0.013020 |  |
|  | p = 0.740941 | p = 0.917397 |  |
| Dicentric | R2 = 0.019737 | R2 = 0.071877 |  |
|  | β = 0.014705 | β = 0.017016 |  |
|  | p = 0.493643 | p = 0.399504 |  |
| Ring | R2 = 0.036559 | R2 = 0.116264 |  |
|  | β = 0.012683 | β = 0.018902 |  |
|  | p = 0.349442 | p = 0.278085 |  |
| Gap | R2 = 0.000985 | R2 = 0.002933 |  |
|  | β = 0.006934 | β = -0.01218 |  |
|  | p = 0.879039 | p = 0.867245 |  |
| Fragment | R2 = 0.001424 | R2 = 0.000159 |  |
|  | β = -0.00696 | β = -0.00251 |  |
|  | p = 0.854788 | p = 0.968953 |  |
| Acentric | R2 = 0.003433 | R2 = 0.002671 |  |
|  | β = 0.008328 | β = -0.00821 |  |
|  | p = 0.776161 | p = 0.873274 |  |
| MLL translocation | R2 = 0.080217 | R2 = 0.16718 |  |
|  | β = -0.06507 | β = -0.11664 |  |
|  | p = 0.160901 | p =0.186926 |  |
| Dupl MLL | R2 = 5.5 x10-5 | R2 = 0.001113 |  |
|  | β = 0.000446 | β = 0.001323 |  |
|  | p = 0.971305 | p = 0.918026 |  |
| Upstream gain | R2 = 0.036204 | R2 = 0.110104 |  |
|  | β = -0.02801 | β = -0.05788 |  |
|  | p = 0.351835 | p = 0.292023 |  |
| Upstream del | R2 = 0.026801 | R2 = 0.028591 |  |
|  | β = -0.00561 | β = -0.00579 |  |
|  | p = 0.42422 | p = 0.599344 |  |
| Downstream gain | R2 = 0.00151 | R2 = 0.000453 |  |
|  | β = 0.001825 | β = 0.00125 |  |
|  | p = 0.850527 | p = 0.947671 |  |
| Downstream del | R2 = 1.58 x10-6 | R2 = 0.002146 |  |
|  | β = 2,03x10-5 | β = 0.00101 |  |
|  | p = 0.99513 | p = 0.88631 |  |
| Del MLL | R2 = 0.006842 | - |  |
|  | β = -0.0025 |  |  |
|  | p = 0.687895 |  |  |
| Total rearrangements of MLL | R2 = 0.055912 | R2 = 0.121002 |  |
|  | β = -0.07986 | β = -0.14449 |  |
|  | p = 0.244834 | p = 0.267872 |  |

**Supplementary Table 3.** The sequence of primers and probes used in RT-qPCR. Gene-F – forward primer, gene-R – reverse primer, gene-Pr – probe. Direction of primers is (5´...3´) and direction of probes is (5´-FAM...3´-BHQ1).

***MLL-AF4*** ^55^

MLL-F2: GATGGAGTCCACAGGATCAGAGT

AF4-R: GAAAGGAAACTTGGATGGCTCA

AF4-Pr: CATGGCCGCCTCCTTTGACAGC

***AML-ETO***  ^55^

AML1-F: CACCTACCACAGAGCCATCAAA

ETO-R: ATCCACAGGTGAGTCTGGCATT

AML1-Pr: AACCTCGAAATCGTACTGAGAAGCACTCCA

***BCR-ABL p190*** ^55^

BCR-m-F: CTGGCCCAACGATGGCGA

ABL-m-R: CACTCAGACCCTGAGGCTCAA

ABL-m-Pr: CCCTTCAGCGGCCAGTAGCATCTGA

***MLL-AF9*** ^56^

MLL1-F: CGCCTCAGCCACCTACTACAG

AF9-R1: TCACGATCTGCTGCAGAATGT

AF9-R2: TGGCAGGACTGGGTTGTTC

AF9-R3: GCTGCTGCTGCTGGTATGAAT

MLL-T1 -Pr: CGCCAAGAAAAGAAGTTCCCAAAACCACT

***c-ABL*** ^57^

c-ABL-F: TGGAGATAACACTCTAAGCATAACTAAAGGT

c-ABL-R: GATGTAGTTGCTTGGGACCCA

c-ABL-Pr: CCATTTTTGGTTTGGGCTTCACACCATT
